# Creatine Kinase Is a Marker of Metabolic Syndrome in Qatari Women With and Without Polycystic Ovarian Syndrome

**DOI:** 10.3389/fendo.2019.00659

**Published:** 2019-09-25

**Authors:** Noora Al-Hail, Alexandra E. Butler, Soha R. Dargham, Ahmed Abou Seif, Stephen L. Atkin

**Affiliations:** ^1^Research Faculty, Weill Cornell Medicine, Doha, Qatar; ^2^Diabetes Research Center, Qatar Biomedical Research Institute, Hamad Bin Khalifa University, Qatar Foundation, Doha, Qatar; ^3^Infectious Disease Epidemiology Group, Weill Cornell Medicine, Doha, Qatar; ^4^Department of Obstetrics, Hamad Medical Corporation, Doha, Qatar

**Keywords:** polycystic ovarian syndrome, creatine kinase, inflammation, obesity, metabolic syndrome

## Abstract

**Objective:** To correlate features of metabolic syndrome with creatine kinase (CK) in women with and without polycystic ovary syndrome (PCOS).

**Design:** Comparative cross-sectional analysis.

**Methods:** Demographic and metabolic data from Qatari women aged 18–40 years from the Qatar Biobank (97 diagnosed with PCOS, 563 controls). The primary outcome was the association between plasma CK and features of metabolic syndrome.

**Results:** CK increased when the waist circumference was >80 cm (*p* < 0.015) and when associated with 2 or more features of the metabolic syndrome (*p* < 0.01). CK correlated with BMI (*p* < 0.003) but not with waist/hip ratio. Overall, CK did not differ between PCOS and controls, rising equally in both as body mass index (BMI) increased. C reactive protein (CRP) was higher in obese PCOS (*P* < 0.05) compared to controls, but did not correlate with CK (*p* > 0.05).

**Conclusion:** CK was associated with an increase in BMI, waist circumference >80 cm and 2 or more features of the metabolic syndrome, in accord with the central role of type II skeletal muscle fibers in energy metabolism and obesity. CK was, however, independent of the PCOS phenotype.

## Introduction

Polycystic ovarian syndrome (PCOS) is the most prevalent endocrine disorder affecting women of reproductive age (1), the prevalence in western populations estimated to be between 6 and 10% depending upon the diagnostic criteria used (2, 3), but is much higher in the Qatari population in the Middle East (4). The condition is characterized by disruption of the menstrual cycle with oligo- or amenorrhea, hyperandrogenism often presenting with acne, seborrhea, and/or hirsutism, and polycystic ovaries (5–7). PCOS is accompanied by a higher incidence of hypertension, insulin resistance and lipid profile abnormalities, resulting in an adverse cardiovascular profile (8–12); a higher prevalence of glucose dysregulation, manifesting as impaired fasting glucose or type 2 diabetes (13, 14); and obesity. However, it is recognized that within the PCOS phenotype there are a number of different androgenic and insulin resistant profiles (15, 16).

Skeletal muscle may contribute to the development of obesity (17, 18), particularly the type II muscle fibers (17, 19). The reason for this is the type II muscle fibers are associated with fatty acid storage in adipose tissue (20), due to their low glucose uptake and reduced mitochondrial oxidation (17, 20). In addition, under normal conditions, skeletal muscle mediates the majority of insulin-stimulated whole-body glucose disposal, and inflammation of muscle with its dysregulation leading to increased insulin resistance is seen in obesity (21). CK is found in high levels in the type II fibers and may be used as a surrogate marker of skeletal muscle fiber number (18, 22). Studies have shown an association between CK and BMI, waist circumference and waist/hip ratio, suggesting that CK is a marker of obesity (18), and with the suggestion that it may identify individuals at risk of obesity (18). The aim of this study was to determine if CK was related to obesity and metabolic syndrome in this Middle Eastern population and whether CK was higher in those women with PCOS, perhaps due to elevated inflammatory markers (23–27), including C-reactive protein (CRP) (28).

## Materials and Methods

### Subjects

This study was undertaken using data obtained from the national Qatar Biobank (QBB) (http://www.qatarbiobank.org.qa). The Qatar Biobank (QBB) was established as a long-term research initiative to benefit the population of Qatar. Adult Qatari men and women years are recruited to undergo a detailed clinical, biochemical and genetic data collection (approved by both the QBB institutional review board (IRB), and by the Ministry of Health Qatar). All participants gave written informed consent for their data and samples to be used in research.

This cross-sectional study involved 660 women between the ages of 18–40 years, 97 with PCOS and 563 control subjects. The diagnosis of PCOS was based upon the NIH criteria of biochemical evidence of hyperandrogenemia (free androgen index >4.5) and oligomenorrhea or amenorrhea; all had information to exclude other confounding diagnoses including thyroid function tests, prolactin, and 17 beta hydroxyprogesterone. All identified PCOS subjects had no documented concurrent illness and were not on any medication. All of the control women reported regular menses, had no biochemical hyperandrogenemia, no documented medical history, nor were they taking any medications. No subject had undertaken exercise in the preceding 3 days prior to sample collection.

Height, weight, waist circumference and body mass index (BMI) were collected according to WHO guidelines and Pulse Wave Velocity (PMV) was measured using VICORDER® PC 400 300E (SMT medical GmbH & Co. KG, Wuerzburg, Germany) as reported previously (4, 29).

All methods of analysis were performed in accordance with the relevant guidelines and regulations with appropriate quality control.

### Collection and Analysis of Blood Samples

After collection, blood samples were immediately processed (within 5 min) and stored at −80°C pending analysis. General chemistry assays and creatine kinase were analyzed by the National Center for Cancer Care, Doha, Qatar, using a Roche Cobas analyzer (Roche Diagnostics, PO Box 50457, Indianapolis, USA), whilst immunoassays were performed for TSH, prolactin, insulin, testosterone, C reactive protein (CRP), DHEAS, and SHBG using an Abbott Architect analyzer (Abbott Laboratories, Abbott Park, Illinois, USA) using the manufacturer's recommended protocol. The functional sensitivity of the testosterone assay was 0.49 nmol/L with intra- and inter-assay coefficients of variation for the assay of 10.0 and 11.3%, respectively. The free androgen index (FAI) was calculated as the total testosterone × 100/SHBG. Serum insulin was assayed using an Abbott Architect analyzer. The analytical sensitivity of the insulin assay was 2 μU/ml, the coefficient of variation was 6%, and there was no stated cross-reactivity with proinsulin. Plasma glucose was measured using a Roche Cobas analyzer. The coefficient of variation for the assay was 1.2% at a mean glucose value of 5.3 mmol/L during the study period. The insulin resistance was calculated using the HOMA method [HOMA-IR = (insulin × glucose)/22.5]. Data were supplied to Weill Cornell Medicine Qatar biostatistics (WCMQ) unit from the QBB in an anonymous coded manner that had been approved by the WCMQ IRB.

The BMI of each subject was stratified according to widely accepted criteria: normal <25 kg/m^2^, overweight 25–29.9 kg/m^2^, obese >30 kg/m^2^.

### Data Analysis

The level of significance was set at 5%. Data trends were visually and statistically evaluated for normality. Independent *T*-tests were applied on normally distributed data, while non-parametric tests (Mann Whitney U) were applied on data that violated the assumptions of normality when tested using the Kolmogorov-Smirnov Test. Statistical analysis was performed using SPSS for Windows, version 24.0. Correlations between CK and demographic parameters were undertaken with Pearson's coefficient, and between CK and CRP with Spearman's coefficient.

## Results

### Demographic Data

The PCOS and control groups were matched for age (Table 1). All subjects were non-diabetic.

**Table 1:** Demographic details for 660 women between the ages of 18–40 years, comparing normal subjects with women defined as having polycystic ovary syndrome by NIH criteria (free androgen index >4.5 and/or a total testosterone >2.7 nmol/l, with irregular menses).

	**Control (*N* = 563)**	**PCOS (*N* = 97)**	***P*-value^**a**^**
	**Mean (*SD*)**	**Mean (*SD*)**	
Age (years)	29.30 (6.00)	28.13 (5.84)	0.076
High Density lipoprotein (mmol/l)	1.57 (0.36)	1.32 (0.3)	<0.001
Systolic blood pressure (mmHg)	104.71 (10.97)	108.69 (10.92)	0.003
Diastolic blood pressure (mmHg)	68.83 (9.33)	72.64 (8.06)	0.001
BMI (kg/m^2^)	26.47 (6.14)	31.25 (10.04)	<0.001
WBC	6.65 (1.73)	7.52 (1.92)	<0.001
Cholesterol (mmol/l)	4.67 (0.71)	4.76 (0.77)	0.236
FPG (mmol/l)	4.75 (0.38)	5.02 (0.95)	<0.01
	**Median (range)**	**Median (range)**	***P*****-value**^**b**^
Creatine Kinase IU/L	62 (1,811)	65 (223)	0.562
Weight (Kg)	65.4 (109.1)	78.8 (72.3)	<0.001
Waist (cm)	77.0 (82.0)	88.0 (57.0)	<0.001
Waist/Hip ratio	0.73 (0.44)	0.79 (0.40)	<0.001
HbA1c (%)	5.2 (9.4)	5.4 (6.6)	<0.001
Insulin (μIU/ml)	7.90 (87.00)	13.00 (110.00)	<0.001
Insulin resistance (HOMA-IR)	1.8 (263.8)	2.6 (32.1)	<0.001
Low density lipoprotein (mmol/l)	2.8 (5.0)	2.9 (3.2)	0.652
Testosterone (nmol/l)	1.1 (2.3)	1.7 (5.8)	<0.001
C-reactive protein (mmol/l)	5.0 (50.0)	5.0 (36.0)	<0.001
FAI	1.52 (3.87)	5.86 (15.43)	<0.001
Pulse wave velocity	9.7 (2.3)	10.0 (1.9)	0.03

a T-test used.

b *Mann-Whitney test*.

The numbers of control and PCOS women in the normal BMI group were 279 (94.9%) and 15 (5.1%), respectively. The numbers of controls and PCOS women in the overweight group were 197 (90.8%) and 20 (9.2), respectively. The number of control and PCOS in the obese group were 165 (79.3%) and 43 (20.7%), respectively.

### Creatine Kinase and Metabolic Syndrome (Figure 1)

The parameters of metabolic syndrome were defined by that of the International Diabetes Federation (30); waist circumference >80 cm, HDL <1.3 mmol/l (50 mg/dl), blood pressure >130/85 mmHg, triglycerides >1.7 mmol/l (150 mg/dl), fasting plasma glucose 5.6 mmol/l (11 mg/dl). When waist circumference less than or greater than 80 cm was compared [median (interquartile range; IQR)] CK was significantly increased (59(34) vs. 65(38) IU). Combining the remaining metabolic syndrome parameters (Table 2), CK showed no difference between 0 and 1 parameters [59(32) and 60(37)IU] but increased significantly when 2 or more parameters were combined [70(40) and 70(35) IU, respectively].

**Figure 1:**
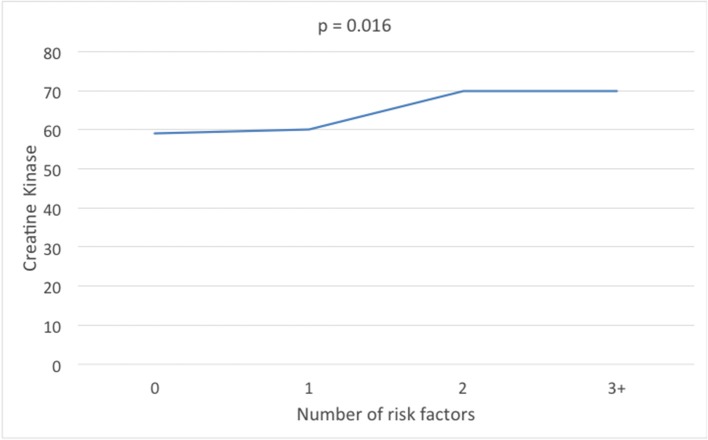
Creatine kinase levels according to the number of metabolic syndrome parameters of high density lipoprotein, triglycerides, fasting plasma glucose, and blood pressure.

**Table 2:** Categorization of the differing metabolic syndrome parameters.

	***N***	**%**
**Waist Circumference and HDL**		
Low WC (<80) and low HDL (<1.29)	57	7.70
Low WC (<80) and high HDL (>1.29)	364	49.00
High WC (>80) and low HDL (<1.29)	138	18.60
High WC (>80) and high HDL (>1.29)	184	24.80
**Waist Circumference and SBP**		
Low WC (<80) and low SBP (<130)	419	56.20
Low WC (<80) and high SBP (>130)	4	0.50
High WC (>80) and low SBP (<130)	313	42.00
High WC (>80) and high SBP (>130)	9	1.20
**Waist Circumference and DBP**		
Low WC (<80) and low DBP (<85)	414	55.60
Low WC (<80) and high DBP (>85)	9	1.20
High WC (>80) and low DBP (<85)	303	40.70
High WC (>80) and high DBP (>85)	19	2.60
**Waist Circumference and Glucose**		
Low WC (<80) and low glucose (<5.60)	396	53.30
Low WC (<80) and high glucose (>5.60)	25	3.40
High WC (>80) and low glucose (<5.60)	268	36.10
High WC (>80) and low glucose (>5.60)	54	7.30

*The different metabolic syndrome parameters were defined as high WC (≥80), low HDL (≤1.29), high SBP (>130), high DBP (>85), and low glucose (≥5.6). Each of these five parameters were coded as 0 (not meeting the mentioned criteria) and 1 (for meeting the mentioned criteria). The metabolic syndrome score was a summation of these five parameters. To assess if a significant difference existed between the levels of metabolic syndrome, we used the Kruskall-Wallis test (CK was not normally distributed). The risk of higher CK was significantly associated (p = 0.014) with those with a sum of MTBS score of 2 compared to a score of 0*.

There was no overall difference in CK between the PCOS and control subjects when all subjects were included (*p* = ns); nor was there a difference when the subjects were stratified according to BMI (Table 3). BMI increase was reflected in the increased waist circumference (Table 3). CRP was higher in those obese subjects with PCOS compared to controls (*p* < 0.02) but CRP was not associated with CK (Pearson's correlation between CK and CRP *R* = 0.022; *P* = 0.560).

**Table 3:** The relationship between BMI categories to creatine kinase, C-reactive protein (CRP), and Waist circumference in subjects with and without PCOS stratified by body mass index.

**BMI**	**Creatine kinase (IU/L)** **median (range)**			**CRP (mg/L) median (range)**			**Waist circumference (cm)** **median (range)**		
	**Control**	**PCOS**	***P*-value^**a**^**	**Control**	**PCOS**	***P*-value^**a**^**	**Control**	**PCOS**	***P*-value^**a**^**
Normal	60 (1,468)	55 (151)	0.359	5.0 (37.0)	5.0 (11.0)	0.167	69.0 (29.0)	70.0 (25.0)	0.175
Overweight	63 (1,811)	67 (179)	0.205	5.0 (50.0)	5.0 (11.0)	0.077	78.0 (28.0)	82.5 (30.0)	0.005
Obese	66 (290)	65 (223)	0.794	5.0 (44.0)	9.0 (36.0)	0.020	89.0 (53.0)	95.0 (38.0)	0.011

The numbers of control and PCOS women in the normal BMI group were 279 (94.9%) and 15 (5.1%), respectively. The numbers of controls and PCOS women in the overweight group were 197 (90.8%) and 20 (9.2), respectively. The number of control and healthy women in the obese group were 165 (79.3%) and 43 (20.7%), respectively.

a Kruskal-Wallis test.

### Pulse Wave Velocity (PWV)

Pulse wave velocity was significantly higher in PCOS compared to controls (Table 1) but using Spearman's correlations, CK and PWV were not significantly correlated. For the overall sample (Control + PCOS), R = −0.021 (*P* = 0.576). Taking the PCOS sample alone, *R* = 0.035 (*P* = 0.741); for the Control sample alone, R = −0.023 (*P* = 0.608).

## Discussion

This study demonstrates that, in this Qatari population, CK was associated with BMI, waist circumference and metabolic syndrome parameters, but that PCOS did not exacerbate the CK levels as they did not differ between PCOS and controls. These data are in accord with a previous study that showed an association of CK levels with BMI and waist circumference (18) though in that study an association with waist hip ratio was found that was not seen here (18). This may be due to the racial mix of participants in that study compared to the homogeneous Qatari population studied here. However, in this study we showed that using the IDF definition of metabolic syndrome (30) with a waist circumference of 80 cm and 2 or more additional features that CK was significantly increased above this cut off. Because of this association, we sought to determine if CK was related to other components of the metabolic syndrome and showed that 2 or more parameters of the metabolic syndrome were associated with an increase in CK. Others have suggested that CK may be a marker for obesity (17–19, 31) given the correlation with BMI and waist circumference. However, waist circumference is a surrogate marker for cardiovascular disease, more so than BMI (32, 33), and the association with the additional composite measures of metabolic syndrome suggests that CK may be a surrogate marker for obesity and for cardiovascular risk.

The prevalence of metabolic syndrome has been shown to be higher in women with PCOS compared to control populations, with an overall pooled odds ratio of 2.09 in a recent meta-analysis (34), with a low high density lipoprotein (HDL), and high waist circumference being the most common components found. As noted above, given that waist circumference is a surrogate marker for cardiovascular disease, more so than BMI (32, 33), and the association of a low HDL with cardiovascular disease, the evidence suggests that these issues need to be addressed in prevention strategies (34).

CRP is an inflammatory marker that is associated with cardiovascular risk (28, 35) and the significantly elevated levels in PCOS seen in this study are well-recognized (28). CRP was not, however, associated with creatine kinase, suggesting that the raised CK was likely not due to an increase in the inflammatory process, though other markers need to be measured to confirm this observation.

Pulse wave velocity reflects arterial stiffness that correlates well to cardiovascular risk. In young subjects (mean age 31 years) it has been suggested that this increased arterial stiffness may relate to greater blood pressure variability (36) that may in turn reflect increased cardiovascular risk. An increase in pulse wave velocity has been previously reported in PCOS (4). However, in this study, pulse wave velocity did not correlate with CK, indicating, as expected, that the CK changes were not due to endothelial dysfunction.

CK is mainly derived from skeletal muscle and, in particular, the type II fibers that are associated with fatty acid storage in adipose tissue (20), due to their low glucose uptake and reduced mitochondrial oxidation (17, 20); it is of note that antioxidant supplementation seems to improve the reproductive outcome in PCOS patients (37, 38). Given that CK has been associated with obesity and insulin resistance (19, 39, 40), both features of the metabolic syndrome, then it is not surprising that the association was found in this group of subjects; however, the PCOS subjects here were even more insulin resistant with a higher BMI and waist circumference and it may have been expected that the CK would have been higher than controls, though this was not found. Thus, there appeared to be a threshold above which (3 or more metabolic syndrome parameters) CK did not show a further rise.

The strengths of the study are the use of biobank data and samples, that were collected in a highly standardized way, and that the study was done on a general population of Qatari women, that would have reduced any sex bias, and with relatively large numbers. As the subjects had not performed any exercise for 3 days prior to sampling, then this should have circumvented any exercise-induced CK rise. Whilst PCOS is recognized as being a heterogeneous condition, all of the subjects fulfilled the NIH criteria and in sufficient numbers to make the findings robust. Limitations of this study are that this was a cross-sectional retrospective study, with no direct measure of abdominal adipose tissue and with only CRP as an inflammatory marker, and a prospective interventional design would have been more powerful; also, the use of biobank data and samples limited the available data and sample types, preventing us from establishing the origin of the plasma CK. We did not have access to muscle biopsy tissue that may have identified the origin of the CK differences.

In conclusion, CK was associated with an increase in BMI, waist circumference >80 cm and 2 or more features of the metabolic syndrome in accord with the central role of type II skeletal muscle fibers in energy metabolism and obesity. This effect was seen independent of the PCOS phenotype.

## Data Availability Statement

The datasets generated for this study are available on request to the corresponding author.

## Ethics Statement

This study was undertaken using data obtained from the national QBB (http://www.qatarbiobank.org.qa). The Qatar Biobank (QBB) was established as a long-term research initiative to benefit the population of Qatar. Adult Qatari men and women years are recruited to undergo a detailed clinical, biochemical, and genetic data collection (approved by both the QBB institutional review board (IRB), and by the Ministry of Health Qatar). All participants gave written informed consent for their data and samples to be used in research in accordance with the Declaration of Helsinki.

## Author Contributions

NA-H drafted the first version, contributed, and approved the manuscript. AB and AA proof read the manuscript, contributed, and approved the manuscript. SD performed the statistical analysis, contributed, and approved the manuscript. SA conceived the study, performed data collation, and approved the manuscript.

### Conflict of Interest

The authors declare that the research was conducted in the absence of any commercial or financial relationships that could be construed as a potential conflict of interest.
